# Prevalence of Chiari malformation type 1 is increased in pseudohypoparathyroidism type 1A and associated with aberrant bone development

**DOI:** 10.1371/journal.pone.0280463

**Published:** 2023-01-20

**Authors:** Neetu Krishnan, Patrick McMullan, Qingfen Yang, Alexzandrea N. Buscarello, Emily L. Germain-Lee

**Affiliations:** 1 Department of Pediatrics, Division of Pediatric Endocrinology & Diabetes, University of Connecticut School of Medicine, Farmington, Connecticut, United States of America; 2 Albright Center, Division of Pediatric Endocrinology & Diabetes, Connecticut Children’s, Farmington, Connecticut, United States of America; 3 Department of Reconstructive Sciences, Center for Regenerative Medicine and Skeletal Development, University of Connecticut School of Dental Medicine, Farmington, Connecticut, United States of America; Medical College of Wisconsin, UNITED STATES

## Abstract

**Background:**

Albright hereditary osteodystrophy (AHO) is caused by heterozygous inactivating mutations in *GNAS*. Patients with maternally-inherited mutations develop pseudohypoparathyroidism type 1A (PHP1A) with multi-hormone resistance and aberrant craniofacial and skeletal development among other abnormalities. Chiari malformation type 1 (CM1), a condition in which brain tissue extends into the spinal canal when the skull is too small, has been reported in isolated cases of PHP1A. It has been hypothesized to be associated with growth hormone (GH) deficiency. Given the adverse clinical sequelae that can occur if CM1 goes unrecognized, we investigated the previously undetermined prevalence of CM1, as well as any potential correlations with GH status, given the known increased prevalence of GH deficiency in PHP1A. We also investigated these metrics for low lying cerebellar tonsils (LLCT), defined as tonsillar descent less than 5 mm below the foramen magnum. In addition, we investigated possible correlations of CM1/LLCT with advanced hand/wrist bone ages and craniofacial abnormalities known to occur in PHP1A to determine whether premature chondrocyte differentiation and/or aberrant craniofacial development could be potential etiologies of CM1/LLCT through both human studies and investigations of our AHO mouse model.

**Methods:**

We examined patients with PHP1A in our clinic and noticed CM1 more frequently than expected. Therefore, we set out to determine the true prevalence of CM1 and LLCT in a cohort of 54 mutation-confirmed PHP1A participants who had clinically-indicated brain imaging. We examined potential correlations with GH status, clinical features, biological sex, genotype, and hand/wrist bone age determinations. In addition, we investigated the craniofacial development in our mouse model of AHO (*Gnas* E1+/-m) by histologic analyses, dynamic histomorphometry, and micro-computerized tomographic imaging (MCT) in order to determine potential etiologies of CM1/LLCT in PHP1A.

**Results:**

In our cohort of PHP1A, the prevalence of CM1 is 10.8%, which is at least 10-fold higher than in the general population. If LLCT is included, the prevalence increases to 21.7%. We found no correlation with GH status, biological sex, genotype, or hand/wrist bone age. Through investigations of our *Gnas* E1+/-m mice, the correlate to PHP1A, we identified a smaller cranial vault and increased cranial dome angle with evidence of hyperostosis due to increased osteogenesis. We also demonstrated that there was premature closure of the spheno-occipital synchondrosis (SOS), a cartilaginous structure essential to the development of the cranial base. These findings lead to craniofacial abnormalities and could contribute to CM1 and LLCT development in PHP1A.

**Conclusion:**

The prevalence of CM1 is at least 10-fold higher in PHP1A compared to the general population and 20-fold higher when including LLCT. This is independent of the GH deficiency that is found in approximately two-thirds of patients with PHP1A. In light of potential serious consequences of CM1, clinicians should have a low threshold for brain imaging. Investigations of our AHO mouse model revealed aberrant cranial formation including a smaller cranium, increased cranial dome angle, hyperostosis, and premature SOS closure rates, providing a potential etiology for the increased prevalence of CM1 and LLCT in PHP1A.

## Introduction

Albright hereditary osteodystrophy (AHO) is a disorder caused by heterozygous inactivation of *GNAS*, which encodes the *α*-subunit of the stimulatory G protein (G*α*_s_) and couples heptahelical receptors to stimulate adenylyl cyclase [1–7]. AHO is classically associated with a constellation of skeletal manifestations that include shortened adult stature, brachydactyly, brachymetacarpia, and the formation of subcutaneous ossifications [8], for review [1–5, 7, 9, 10]. However, AHO has also been associated with craniofacial abnormalities that include midface hypoplasia, craniosynostosis, dental and jaw abnormalities, and cranial hyperostosis [2, 4, 10–12]. In addition to these skeletal features, patients with *GNAS* mutations on the maternally inherited allele develop pseudohypoparathyroidism type 1A (PHP1A), in which patients exhibit obesity and resistance to multiple hormones requiring G*α*_s_-coupled receptors [such as parathyroid hormone (PTH), thyrotropin (TSH), luteinizing hormone (LH), follicle-stimulating hormone (FSH), calcitonin, glucagon, and growth hormone releasing hormone (GHRH)] [9, 13–18]. In contrast, patients with paternally-inherited *GNAS* mutations develop pseudopseudohypoparathyroidism (PPHP) [19] and exhibit classic AHO skeletal features but do not exhibit severe obesity [20] or hormonal resistance, [for review [1–5, 7, 9, 10]]. It has been established in both humans and mouse models [1] that the metabolic and hormonal distinctions between PHP1A and PPHP are secondary to partial paternal imprinting of *GNAS* in specific tissues, many of which are endocrine, such as the renal proximal convoluted tubules, thyroid, gonads, and pituitary [13–15, 21–24]. In addition, we have found in both human and mouse models that regulation of bone remodeling and bone mineral density may be impacted by imprinting as well [25–27].

Over the course of the past two decades, our clinic has evaluated several hundred mutation-confirmed patients with PHP1A and has identified through both clinical work-ups and routine brain imaging that a substantial cohort of PHP1A patients displayed features of Chiari Malformation Type 1 (CM1). First described by Hans van Chiari in 1891 [28, 29], CM1 is defined as a downward displacement of the cerebellar tonsils below the foramen magnum of at least 5 mm [30, 31]. This condition, in which brain tissue extends into the spinal canal, can occur when part of the skull is misshapen or smaller than normal, thereby pressing on the brain and forcing it downward. Chiari malformation type 1 develops as both the brain and skull are growing, and as a result, signs and symptoms may not occur until late in childhood or into adulthood. Within the general population, it is estimated that the true prevalence of CM1 is less than 1 percent [30, 32, 33]. It has been well documented that CM1 can potentially cause significant morbidity and mortality, thereby emphasizing the importance of early diagnosis [34, 35]. Clinical symptoms associated with CM1 vary among adult and pediatric patients; in adults, common symptoms include headache, vertigo, dysphagia, and other signs of craniovertebral instability [36], whereas in very young children, symptoms may manifest more diffusely as central sleep apnea, feeding difficulties, irritability, and failure to thrive [37, 38]. Low lying cerebellar tonsils (LLCT), sometimes referred to as benign cerebellar tonsillar ectopia, are a distinct radiographic finding from CM1 and are defined as tonsillar descent less than 5 mm below the foramen magnum [39–41]. Although LLCTs are often clinically asymptomatic, a recent case report detailed a 5-month-old patient with LLCT that became symptomatic and further progressed an additional 2 cm below the foramen magnum over a span of a few years, later requiring surgical correction [42]. Therefore, while we understand that LLCT usually is not clinically symptomatic, it appears prudent to consider this as a pertinent finding in the development of CM1.

While it has been over a century since CM1 was first described, the underlying etiology of this radiologic finding remains unknown. Studies in the general population have identified a number of potential contributing causes that include aberrant cranium development that can be due to craniosynostosis or hyperostosis [38, 43–46] or secondary to growth hormone (GH) deficiency [47, 48]. Among these etiologies, the most commonly observed skeletal anomaly involves malformations in the posterior cranial fossa [37, 43, 49, 50] due to underdevelopment of the occipital bone leading to overcrowding and subsequent tonsillar herniation. Additional reports have also identified an association of CM1 with aberrant fusion of the spheno-occpital synchondrosis (SOS) [37, 51]. The SOS is a cartilaginous structure that serves an essential role in the development of the cranial base. Accelerated closure of the SOS in humans and mice have also been correlated with aberrant cranial patterning and the development of midface hypoplasia [52]. Furthermore, prior reports have implicated similarities to cranial synchondroses to that of the epiphyseal plates of long bones due to the cell types harboring these regions, as well as the presence of highly conserved signaling pathways influencing cellular differentiation and bone development [53].

In addition to structural cranial abnormalities, studies in the general population have also suggested that CM1 development can be secondary to GH deficiency [47, 48] with some studies finding CM1 formation in up to 20% of GH deficient patients [47]. It was hypothesized that GH deficiency can lead to underdevelopment of the posterior fossa, thus leading to CM1 formation [47, 54–56]. According to the International Consensus statement for pseudohypoparathyroidism and related disorders [2, 10], CM1 is mentioned as a possible occurrence in PHP1A based on a small collection of isolated case reports [57–60]. These reports are intriguing given that GH deficiency has been found in approximately 70% of PHP1A patients secondary to GHRH resistance [4, 17, 18]. This is much greater than the frequency of GH deficiency in the general population of 1:3,480 [61]. In alignment with these findings, two recent case reports describe PHP1A patients who developed CM1 and were GH deficient [57, 60]. In both of these isolated reports, the authors hypothesized that the development of CM1 was secondary to inappropriate cartilage growth at the cranial base due to GH deficiency.

In addition, both PHP1A and PPHP patients have rapidly advancing bone ages and lack of pubertal growth spurts secondary to early epiphyseal fusion due to premature chondrocyte differentiation [1, 3, 4, 9, 17, 62, 63]. It is hypothesized that the biallelic expression of *GNAS* in chondrocytes leads to this occurrence in both PHP1A and PPHP patients [4, 64–67]. This raises the question as to whether premature synchondrosis closure could be involved in the etiology of CM1 and LLCT, as the synchondrosis is a structure recognized to be essential for craniofacial development; accelerated closure of this structure in humans and mice has been correlated with the development of midface hypoplasia, a common clinical finding in PHP1A.

Because the true prevalences of CM1 and LLCT in PHP1A patients are not known and because the potential association of these abnormalities with PHP1A would have important clinical implications, we investigated a large cohort of mutation-confirmed PHP1A participants who had been evaluated in our Albright Center, a clinic dedicated to the care of patients with AHO and in which we have evaluated several hundred mutation-confirmed patients from around the world. Within the clinic population, we selected those mutation-confirmed PHP1A patients who also had brain imaging in order to determine the prevalence of CM1/LLCT. We also performed secondary analyses to identify whether there were independent factors that positively correlated with CM1/LLCT development, such as GH status, clinical findings, biological sex, *GNAS* mutation severity, and bone age. On the basis of these clinical observations, we then systematically examined our AHO mouse model generated through the targeted disruption of *Gnas* exon 1 (*Gnas E1+/-*) [22, 26, 68] to evaluate how *Gnas* heterozygous inactivation influences craniofacial development and bone formation. By utilizing various investigative techniques, we systematically analyzed the various hypothesized structural causes of CM1 formation in our mouse model.

## Methods

### Human subjects approval

All patient studies were approved by the Johns Hopkins Medicine Institutional Review Board (includes Kennedy Krieger Institute), Connecticut Children’s Institutional Review Board, and the University of Connecticut School of Medicine Institutional Review Board. Written informed consent was obtained from all participants, or a parent of each participant, along with the signature of an adult witness at the time of consent. Written assent was also obtained when appropriate based on age and emotional/cognitive maturity, again with signature of an adult witness at the time of assent.

### PHP1A participant characteristics: Age, sex, hormonal and biochemical evaluation

**Fig 1:**
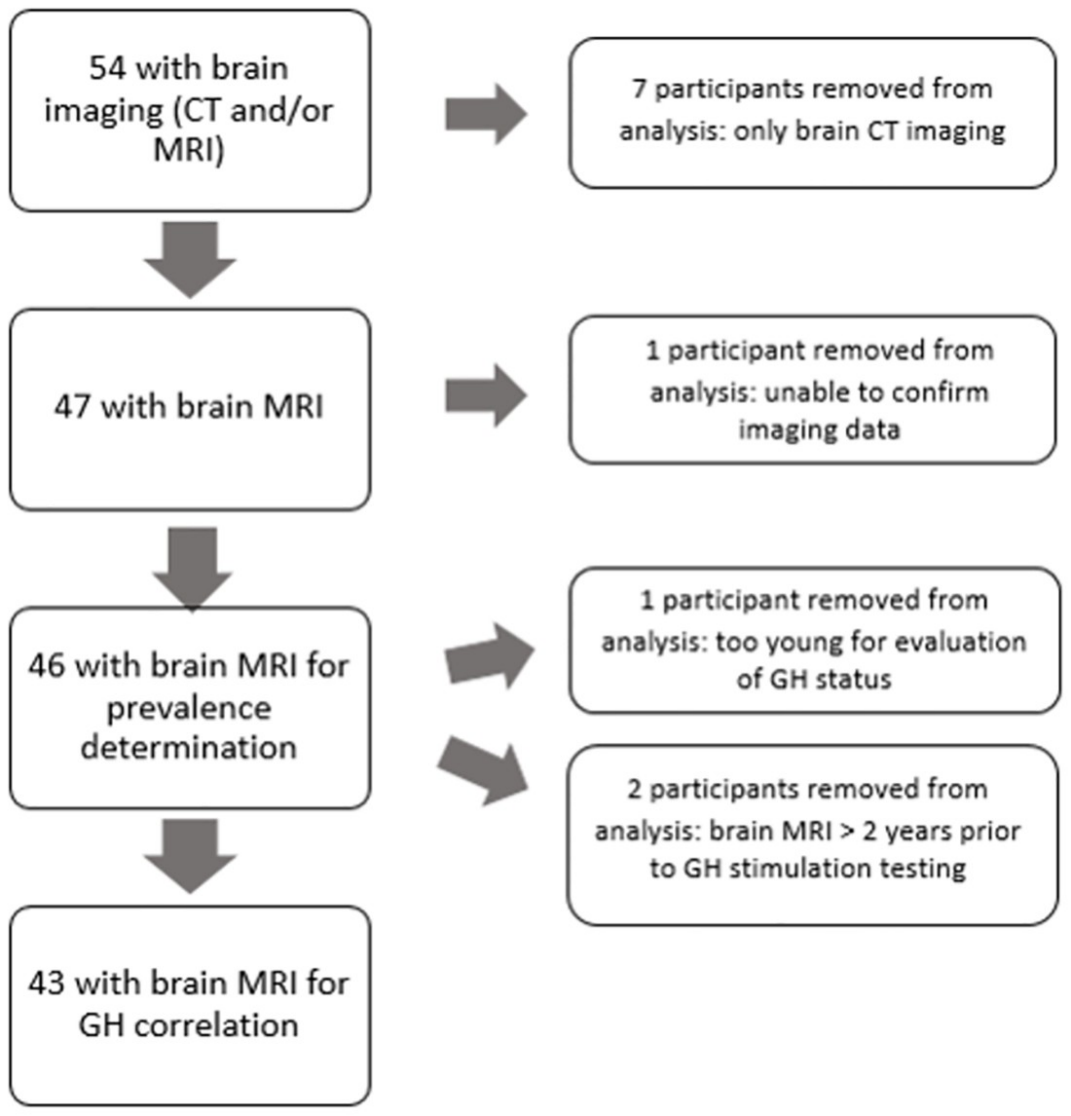
Inclusion/exclusion criteria flowchart. We identified 54 patients with PHP1A and GH deficiency who had brain CT and/or MRI imaging within our cohort. Of these, 47 had a brain MRI performed. One participant was excluded, as we were unable to confirm the imaging data. This resulted in 46 participants for the prevalence calculations. From these, 3 were further excluded, as they were either too young for evaluation of GH status or had a brain MRI >2 years prior to GH stimulation testing. The remaining 43 participants were included in the GH versus CM1/LLCT correlation calculations.

A cohort of 54 mutation-confirmed PHP1A participants were seen by a consistent provider (E.L.G-L.) in the Albright Center at either Johns Hopkins, Kennedy Krieger Institute, or Connecticut Children’s. In addition to mutation confirmation, all 54 participants had a subset of physical features consistent with AHO and evidence of multi-hormone resistance consistent with their diagnosis of PHP1A. All participants had PTH and TSH resistance with most also having GHRH resistance (see Table 1). Laboratory testing was performed by the Johns Hopkins laboratory for all those patients seen at Johns Hopkins and Kennedy Krieger Institute or at Quest Diagnostics laboratory for patients seen at Connecticut Children’s. GnRH resistance was determined clinically by physical examination and clinical symptoms. In females this was also done by obtaining menstrual histories, and if amenorrhea or oligomenorrhea was present, then the participant met criteria for being GnRH deficient. Of the 46 participants with brain MRIs performed (see Fig 1), 27 were females and 19 were males. The mean age of participants at the time of brain MRI imaging was 9.50 years. Adults were defined as participants over the age of 18 years. Twenty-nine out of 45 participants were noted to have midface hypoplasia. As midface hypoplasia is not always apparent in early life, we could not assess one patient, as this participant was seen only at a very young age.

**Table 1:** Characteristics of mutation-confirmed PHP1A cohort.

ID #	Sex	Mutation	Age at MRI (yr)	Age at GH testing (yr)	GH deficient	Hormonal Resistance at Imaging	CA at BA (yr)	SD for CA (± yr)	BA (yr)	Advanced BA
**Participants with Chiari 1 Malformation**										
1	F	Exon 5: c.343C>T (p.Pro115Ser)	˟3.4	6.0	NO	PTH, TSH	5.7	±0.7	6.8	NO
2	M	Exon 5: c.348delC	9.7	9.9	YES	PTH, TSH, GHRH	10.1	±0.8	11.5	NO
3	F	Exon 7: c.565-568delGACT	11.3	11.3	YES	PTH, TSH, GHRH	11.0	±1.0	13.0	YES
4	M	Exon 7: c.565-568delGACT	4.5	3.3	YES	PTH, TSH, GHRH	4.5	±0.7	6.0	YES
5	M	Exon 10: c.793C>T (p.Arg265Cys)	5.4	5.2	YES	PTH, TSH, GHRH	6.0	±0.8	11.0	YES
**Participants with Low Lying Cerebellar Tonsils**										
6	F	Exon 1:c.85C>T (p.Gln29*)	7.2	7.0	YES	PTH, TSH, GHRH	7.0	±0.8	11.0	YES
7	F	Exon 6: c.470-473del	8.9	8.7	YES	PTH, TSH, GHRH	BA not avail-able	n/a	Pre-mature fusion of distal femurs noted at age 4	n/a
8	M	Exon 7: c.565-568delGACT	12.5	11.0	YES	PTH, TSH, GHRH	13.5	±0.8	13.5	NO
9	F	Exon 10: c.730A>T (p.Ile244Phe)	10.4	9.8	YES	PTH, TSH, GHRH	9.9	±1.0	13.5	YES
10	F	Exon 12: c.1024C>T (p.Arg342*)	˟31.7	43.2	NO	PTH, TSH	ᵟAdult	n/a	ᵟAdult	n/a
**Participants with Neither CM1 Nor Low Lying Cerebellar Tonsils**										
11	F	Exon 1: c.3G>A (p.Met11Ile)	˟0.1	N/Aˆ	N/Aˆ	PTH, TSH	BA not avail-able	n/a	BA not avail-able	n/a
12	M	Exon 1: c.21insT	˟19.1	21.1	YES	PTH, TSH, GHRH	ᵟAdult	n/a	ᵟAdult	n/a
13	F	Exon 1: c.21insT	21.1	18.0	YES	PTH, TSH, GHRH, LH/FSH	ᵟAdult	n/a	ᵟAdult	n/a
14	F	Exon 1: c.85C>T (p.Gln29*)	10.4	10.3	YES	PTH, TSH, GHRH	10.3	±0.9	13.0	YES
15	M	Exon 1: c.91C>T (p.Gln31*)	6.0	6.0	YES	PTH, TSH, GHRH	9.3	±0.8	8.0	NO
16	F	Exon 1: c.103C.T (p.Gln35*)	19.6	19.6	YES	PTH, TSH, GHRH, LH/FSH	ᵟAdult	n/a	ᵟAdult	n/a
17	F	Exon 1: c.124C>T (p.Arg42Cys)	˟0.5	7.4	NO	PTH, TSH	7.8	±0.8	8.8	NO
18	F	Exon 5: c.317T>C (p.Ile106Thr)	˟3.5	7.9	YES	PTH, TSH, GHRH	7.0	±0.8	7.8–8.8ᶿ	n/a‡
19	F	Exon 5: c.348dupC	4.9	4.8	YES	PTH, TSH, GHRH	4.9	±0.9	5.0	NO
20	F	Exon 5: c.348delC	3.1	3.1	YES	PTH, TSH, GHRH	3.3	±0.6	3.5	NO
21	M	Exon 5: c.352 G>T (p.Glu118*)	2.5	2.2	NO	PTH, TSH	4.0	±0.6	4.5	NO
22	F	Exon 5: c.366delC	4.7	4.6	YES	PTH, TSH, GHRH	5.3	±0.9	5.8	NO
23	F	Exon 5: c.393 C>T (Ile131Ile); Exon6: c.493C>T (Arg165Cys)	3.0	3.0	YES	PTH, TSH, GHRH	BA not avail-able	n/a	BA not avail-able	n/a
24	F	Exon 5: c.393C>T (Ile131Ile)	˟15	5.0	YES	PTH, TSH, GHRH	BA not avail-able	n/a	BA not avail-able	n/a
25	F	Exon 6: c.468T>G (p.Asp156Glu)	9.7	9.2	YES	PTH, TSH, GHRH	8.7	±0.9	13.5	YES
26	M	Exon 6: c.468T>G (p.Asp156Glu)	8.0	7.9	YES	PTH, TSH, GHRH	7.1	±0.8	14.5	YES
27	M	Exon 7: c.565-568delGACT	13.1	12.5	YES	PTH, TSH, GHRH	12.4	±0.8	16.0	YES
28	M	Exon 7: c.565-568delGACT	11.4	11.3	YES	PTH, TSH, GHRH	11.3	±0.8	14.0	YES
29	M	Exon 7: c.565-568delGACT	9.9	9.8	YES	PTH, TSH, GHRH	9.9	±0.8	12.5	YES
30	F	Exon 7: c.565-568delGACT	12.0	10.7	YES	PTH, TSH, GHRH	9.9	±1.0	13.5	YES
31	M	Exon 7: c.565-568delGACT	˟3.1	4.3	YES	PTH, TSH, GHRH	4.3	±0.7	4.3	NO
32	F	Exon 7: c.565-568delGACT	12.2	11.7	YES	PTH, TSH, GHRH	11.8	±0.8	15.0	YES
33	F	Exon 7: c.568-569delTA	7.9	7.8	YES	PTH, TSH, GHRH	7.9	±0.8	8.8	NO
34	M	Del Exon 7–13	4.8	4.3	YES	PTH, TSH, GHRH	5.0	±0.8	5.0–6.0ᶿ	NO
35	M	Exon 9: c.701G>A (p.Trp234*)	˟1.2	4.5	YES	PTH, TSH, GHRH	4.7	±0.9	4.0	NO
36	M	Exon 9: c.710G>A (p.Cys237Tyr)	˟10.8	11.4	YES	PTH, TSH, GHRH	11.1	±0.8	13.0	YES
37	F	Intron 9: c.719-2A>C	˟33.0	12.4	YES	PTH, TSH, GHRH, LH/FSH	12.3	±0.8	13.5	NO
38	F	Exon 10: c.772C>T (p.Arg258Trp)	23.2	22.9	YES	PTH, TSH, GHRH	ᵟAdult	n/a	ᵟAdult	n/a
39	M	Exon10: c.784C>T (p.Gln262*)	11.0	11.1	YES	PTH, TSH, GHRH	10.7	±0.9	11.5	NO
40	F	Exon 12: c.1006C>T (p.Arg336Trp)	12.3	12.1	YES	PTH, TSH, GHRH	11.7	±1.0	13.5	NO
41	M	Exon 13: c.1039-2delA	12.7	12.8†	YES	PTH, TSH, GHRH	12.4	±0.8	13.0	NO
42	F	Exon 13: c.1083insC	7.9	6.7	YES	PTH, TSH, GHRH	8.4	±0.7	8.0	NO
43	F	Exon 13: c.1100insA	˟2.1	3.2	YES	PTH, TSH, GHRH	3.0	±0.5	3.0–3.5ᶿ	NO
44	M	Exon 13: c.1100insA	4.4	4.1	YES	PTH, TSH, GHRH	4.0	±0.7	8.0–9.0ᶿ	YES
45	M	Exon 13: c.1100insA	˟10.3	3.8	YES	PTH, TSH, GHRH	3.0	±0.4	4.5	YES
46	F	Exon 13: c.1174G>A (p.Glu392Lys)	10.8	9.9	YES	PTH, TSH, GHRH	11.0	±1.0	12.0	NO

Abbreviations: CA- chronologic age, BA- bone age, SD- standard deviation, yr- year(s)

º advanced bone age defined as greater than or equal to 2 standard deviations between bone age and chronological age

˟ participants with imaging performed for reasons other than GH deficiency

* stop codon

ᵟ adult defined as participants > 18 years old

ˆ participant was too young for GH testing and accurate assessment of midface hypoplasia evaluation

ᶿ participant with dysharmonic bone age

^‡^ calculated advanced bone age falls within the range of dysharmonic bone age, so removed from analysis

^†^ date at which GH stimulation testing by another provider was attempted but failed due to difficult access

### Bone marker evaluation

Bone formation markers including calcium, phosphorus, alkaline phosphatase, PTH, and 25-OH Vitamin D levels in all participants were also evaluated. We analyzed available data from the time of evaluation up to and including the time of the brain MRI date for each individual participant. C-telopeptide levels were available for a small subset of participants; however there were too few performed for statistical calculations, or these levels were obtained after the brain MRIs were performed, and therefore not included.

### *GNAS* mutation analysis

All PHP1A participants in this study had mutation confirmation of their diagnosis. Peripheral blood from all patients was collected at the Albright Center at the John Hopkins Institute of Clinical and Translational Research (ICTR), the Kennedy Krieger Institute, or Connecticut Children’s. DNA isolation and *GNAS* mutation analyses of the 13 coding exons and all intron/exon boundaries were performed either in the Johns Hopkins DNA Diagnostic Laboratory (http://www.hopkinsmedicine.org/dnadiagnostic/), the Center for Genetic Testing at St. Francis (http://www.sfh-lab.com/), or in a research laboratory (E.L.G-L.) at the Johns Hopkins University School of Medicine [9, 17]. The DNA diagnostic laboratories at both Johns Hopkins and St. Francis are approved by the Clinical Laboratory Improvement Amendments. Types of mutations included missense, nonsense, deletion, insertion, and frameshift within the portion of the *GNAS* gene encoding the *α*-subunit of the stimulatory G protein (G*α*_s_). There was one patient with an intronic mutation within the *GNAS* locus.

For purposes of this analysis, missense mutations were considered mild, while the other mutations were considered severe. Comparisons between type of mutation and presence of CM1 versus LLCT versus neither were analyzed (see Table 1).

### Brain imaging

A total of 54 participants had brain imaging performed, which included MRI and computerized tomography (CT) scans. Imaging was done either at a certified radiology facility near the participant’s home or the facility at which E.L.G-L. saw the participant, which includes either Johns Hopkins (where Kennedy Krieger participants are also imaged) or Connecticut Children’s Medical Center. All of the images were read by board-certified radiologists. For the purpose of our analysis, we excluded any participant with only a brain CT scan performed because MRI is considered the gold standard for diagnosing CM1 [37, 38]. In addition, we only included MRI imaging for which we had full access to the official report and/or actual images for review (see Fig 1). With the exception of 13 participants, all participants underwent brain MRI after they failed GH stimulation testing as per standard of care. These 13 patients had brain MRIs performed by their primary endocrinologists for various clinical reasons such as symptoms of CM1, seizures, concern for precocious puberty, developmental delays, lethargy, central sleep apnea, hearing or memory loss, loss of smell, and evaluation for pituitary lesion. Findings of CM1 or LLCT were documented after reviewing each MRI. The degree of tonsillar herniation was also documented if the information was available. CM1 was defined as cerebellar tonsillar herniation at least 5 millimeters (mm) below the foramen magnum [30, 31, 69]. LLCT was defined as cerebellar tonsillar herniation less than 5 mm below the foramen magnum. Some centers referred to these as low lying cerebellar tonsils, while others described this as cerebellar tonsillar ectopia [39–41].

### Determination of growth hormone status

Of the 46 participants who had brain MRI imaging, 43 participants had GH stimulation testing performed by a well-accepted standard-of-care protocol. All participants were evaluated and assessed to be on appropriate treatment for their hormonal resistances with good control biochemically prior to the GH stimulation testing as well as having had appropriate thyroid function, PTH, and serum calcium levels at the time of GH testing. The majority of GH stimulation testing included arginine-L dopa testing as reported previously [17] performed as per standard clinical protocol [70, 71]. The remainder were performed by arginine-clonidine [71–73], with one by arginine-insulin [71, 73]. GH deficiency was determined according to standard clinical procedure [73]. One participant was too young for GH stimulation testing at the time of our evaluation and therefore not included in the correlation studies. One participant had GH stimulation testing performed many years prior to evaluation but was only seen by us after GH therapy had been completed. Her records revealed thorough documentation of GH deficiency in all provider notes, appropriate dosing of GH treatment, and a robust response to therapy based on her growth curve, but the actual results of the testing were unavailable. She was included in the cohort that we examined. Another participant, for whom IV access was not possible at the time of testing at an outside institution, did not have GH stimulation testing performed. A low serum IGF-1 level was documented, and records revealed poor linear growth prior to GH initiation with a robust response to treatment; therefore, this participant was also included in the analysis. In all participants in which GH testing was performed, the diagnosis of GH deficiency was based on a peak serum concentration of GH less than 7 or 10 ng/ml in children based on the assay utilized at the time [73, 74] and less than or equal to 5 ng/ml in adults [74, 75].

### Radiograph and Dual-energy X-ray absorptiometry (DXA) analysis

All of the participants had radiographs for bone age analyses as part of routine clinical care unless already adults. These included hand/wrist, knee, and foot/ankle X-rays, as the hand/wrist bone age is typically the most advanced in PHP1A and difficult to utilize clinically for assessment of growth potential at the epiphyses [1, 9, 17]. These radiographs were performed at the Albright Center at either the Johns Hopkins Hospital, the Kennedy Krieger Institute, Connecticut Children’s, or Jefferson Radiology (Farmington, CT), or at a radiology facility near the participant’s home with the radiographs sent to one consistent examiner (E.L.G-L.) to read, as with all the others. Bone age of the left hand and wrist was determined according to the Gruelich and Pyle standards [76]. An advanced bone age was defined as a bone age greater than or equal to 2 standard deviations above the mean as per standard of care criteria. The *GNAS* mutation itself leads to premature chondrocyte differentiation (for review [1, 4, 9]) [64–66, 77], which is the etiology of the marked advancement of the hand/wrist bone age in AHO. The advancement is not as great at the knee, presumably secondary to the marked shortening of the phalanges and affected metacarpals whereas long bone shortening is not as typical [1, 4, 9, 17, 67]. For treatment with GH, knee bone ages have been demonstrated to be the most accurate in AHO [1, 4, 9, 17, 67].

Skull X-rays were performed only for standard of care indications and in a total of 4 patients.

DXA scans had been performed on a subset of patients: 0 with CM1, 3 with LLCT, and 5 unaffected as part of a prior research study according to methods described previously [25]. Head bone mineral density (BMD) measurements from these images were evaluated.

### Generation and maintenance of *Gnas* E1+/- mice

All mouse protocols were carried out in accordance with the standards of the UConn Health Animal Care and Use Committee (AP-200074-0623). The generation of mice carrying a targeted disruption of exon 1 of *Gnas (Gnas E1+/-)* was described previously [22]. Mice were maintained on a pure 129SvEv background and were genotyped by PCR analysis. Each 20 μL reaction was performed with 2 μL DNA, 3μL of 10x Standard Taq Reaction Buffer (NEB), 0.1 μL Taq Polymerase (NEB), 0.2ul of 40μM *Gnas* Forward and *Gnas* Reverse, 0.3ul of 40uM *Neo1* primers (sequences below), 1ul of 10mM of dNTP mix (Promega), and 13.1 μL of InVitrogen Ultrapure Distilled Water. Reaction parameters were carried out as follows: 95°C for 5 minutes, 34 cycles of: (1) 95°C for 30 seconds; (2) 60°C for 30 seconds; (3) 72°C for 30 seconds, and 72°C for 5 minutes. All mice that carry a mutant maternal *Gnas* allele are hereafter referred to as *Gnas E1+/-m* and those with a mutant paternal allele as *Gnas E1+/-p*. Wild type mice (*Gnas E1+/+*) are referred to as WT.

**Table:** 

**PCR Oligonucleotide Primer Sequences**		
**Gene**	**Forward (5’ → 3’)**	**Reverse (5’ → 3’)**
*Gnas*	TCGTCCCCTCAGTTGGCCAC	CCTCCCAACAAATCGCACAC
*Neo 1*	GAATTCGCCAATGACAAGAC	

### Radiographic (faxitron) imaging

Radiographic imaging was performed on harvested skull samples of 3 week and 12-week *Gnas E1+/-* and *WT* mice using a Faxitron machine (exposure period of 26 kV for 3 seconds). Measurements of skull length and height were identified using the metrics previously described by Navein *et al*. and calculated using the open source software ImageJ [78].

### Μicrocomputed tomography (MCT) analysis

Analysis of craniofacial parameters were completed on 12-week *Gnas E1+/-* and WT mice using microcomputed tomography (uCT 40; Scanco Medial AG, Bassersdorf, Switzerland). Following euthanasia, skull samples were harvested from *WT* and *Gnas E1+/-* mice, dissected to remove surrounding connective tissue and fixed in 70% Ethanol at 4°C. ΜCT analysis was performed using the μCT50 Scanco Medical scanner with the following parameters: voxel size of 17.2μm, 55kV and 145μA. Three-dimensional reconstructive images were visualized, and linear measurements were performed using the open source software MeshLab and ImageJ. Measurement of foramen magnum area was calculated by using both the Radinsky and Teixeira equations as previously described [79–82]. Measurement of cranial dome angle was identified using MCT images following the metrics previously described by Navein *et al*. [78] (calculated using ImageJ).

### Skeletal whole mount staining

Whole mount staining of P14 days (2 week) old *WT* and *Gnas E1+/-* mice was performed using the methods of *Rigueur* and *Lyons* [83]. Briefly, skull samples were harvested, dissected, and fixed overnight in 95% ethanol. Fixed samples were next submerged in acetone overnight, followed by staining for 24 hours with 0.03% Alcian Blue solution dissolved in 80% ethanol and 20% glacial acetic acid. The specimens were then washed in 95% ethanol overnight and cleared with 2% potassium hydroxide for 2 hours. The samples were stained for 8 hours in a 0.005% Alizarin Red dissolved in 1% potassium hydroxide (KOH). Finally, samples were washed in 2% KOH overnight and then stored in 100% glycerol. Samples were visualized using a dissecting microscope.

### Histology

Following euthanasia by CO_2_ asphyxiation, skull samples from 1 week, 2 week, and 12-week-old mice were harvested, dissected to remove surrounding tissue, and fixed in 10% Neutral Buffered Formalin (NBF) for 3 days at 4°C. Harvested specimens were transferred to PBS for 2 hours and subsequently placed in 30% Sucrose for 48 hours at 4°C. Samples were then embedded into Optimal Cutting Temperature (OCT) compound. Tissue blocks were stored at -20°C until use, and cryosections (10–15μm) were collected using a cryostat tape-transfer system (Section-lab, Hiroshima, Japan) as previously described [84].

### Dynamic histomorphometry using bone mineral labels

Bone formation activity was assessed in 12-week-old *WT* and *Gnas E1+/-* mice by performing alizarin complexone (AC) and calcein double-labeling using methods as previously described [26, 84]. Briefly, *WT* and *Gnas E1+/-* mice received an intraperitoneal injection of alizarin complexone [dose 30mg/kg (Sigma A-3882)] 7 days prior to sacrifice, in addition to an injection of calcein [dose 10mg/kg (Sigma C-0875)] 2 days prior to sacrifice. Non-decalcified calvarial and cranial base sections were rehydrated in PBS for 30 minutes and subsequently stained with calcein blue [30mg/mL calcein blue in 2% NaHCO_3_ in H_2_O pH 7.4 (Sigma M1255)] for 10 minutes to visualize total mineral content, mounted, and cover-slipped using 30% glycerol in PBS. Samples were imaged using a Ziess Axioscan Z1 high-speed automated image acquisition system (Cat#440640-9903-000) and a high-resolution camera (AxioCam HRm). Dynamic histomorphometry parameters including mineral apposition rate (MAR), mineralizing surface to bone surface (MS/BS), and bone formation rate (BFR) were calculated using the semi-automated open source CalceinHisto bone histomorphometry software [85].

### Cranial base synchondrosis histomorphometry

Histomorphometric analysis of the cranial base of P7 and P14 *WT* and *Gnas E1+/-* mice was performed by utilizing a modified multiplex fluorescence microscopy method as described by Dyment *et al*. [84]. For these studies, mice were administered one intraperitoneal injection of EdU (50 mg/kg body weight) 4 hours prior to tissue harvest. Non-decalcified sections were rehydrated in PBS for 30 minutes and subsequently stained with calcein blue for 10 minutes, mounted, and coverslipped using 30% glycerol in PBS. Following image acquisition, slides were stained using alkaline phosphatase (ALP) enzyme histochemistry (methods described [84]) for 5 minutes to visualize hypertrophic chondrocytes and bone lining osteoblasts. Sections were washed in PBS for 15 minutes and were subsequently incubated in an EdU staining cocktail containing 100mM Tris buffer (pH 7.4), 2mM CuSO4, 10mM Ascorbic Acid, and 5uM Sulfo-FITC488-Azide for 30 minutes to identify proliferating cells [86]. Sections were washed again for 15 minutes in 100mM Tris Buffer (pH 7.4), mounted in 50% glycerol/PBS with DAPI, and coverslipped for image acquisition. Following image acquisition, samples were washed for 15 minutes in deionized water and were stained for 5 minutes in Weigert’s Iron Hematoxylin to visualize nuclei, washed in deionized water for 5 minutes, stained in 0.2% fast green solution for 2 minutes, rinsed in 1% acetic acid solution for 2 minutes, and stained in 0.1% safranin O solution for 1 minute. Following image acquisition, one composite image stack containing the channel images from each staining round was assembled using Photoshop as previously described [84]. Quantification of spheno-occipital synchondrosis length on composite images were calculated using ImageJ. Additionally, quantification of specific chondrocyte zone lengths including the hypertrophic zone (defined as length of SOS-containing ALP+ hypertrophic chondrocytes) as well as the resting/proliferative zone (defined as ALP-negative region of SOS) were measured using ImageJ. Finally, the percentage of EdU+ chondrocytes compared to the total number of chondrocytes residing within the resting/proliferative zone were quantified using ImageJ.

### Lambdoid and interparietal suture histomorphometry

Histomorphometric analyses of the lambdoid and interparietal sutures of P7 *WT* and *Gnas E1+/-* mice were performed utilizing non-decalcified sagittal tissue sections of the skull stained with calcein blue for 10 minutes to visualize total mineral content. Slides were mounted and cover-slipped using 30% glycerol in PBS and imaged. Quantification of suture length was determined using ImageJ.

### Statistics

All statistical analyses were performed using Graphpad Prism Version 9, with *p*-values < 0.05 considered to be statistically significant. Chi square analysis was used to determine the relationship between GH deficiency, biological sex, and bone age advancement with CM1 or LLCT. This analysis was also used to look at the relationship between the skull bone mineral densities from DXA scans between patients with LLCT and those with normal brain MRIs. The standard chi square formula was used where “O” is the observed value and “E” is the expected value [87]. For all mouse analyses observed at one discrete time point, data obtained from WT, *Gnas E1+/-p*, and *Gnas E1+/-m* were analyzed by a one-way ANOVA with a post hoc Tukey test for multiple comparisons. For all mouse analyses compared at multiple time points, a two-way ANOVA with a post hoc Tukey test for multiple comparisons was utilized. For human analyses, the latter ANOVA was utilized. Each *n* value refers to the number of mice, human participants, or human laboratory values for a given parameter. All data points were included in the data analysis unless otherwise stated. No samples were excluded. Raw data mouse data are included within the manuscript and in the figures/figure legends and all significant human data are included in the manuscript and the table/table legend.

## Results

### Prevalence of Chiari Type 1 malformation as related to GH status, bone-related biochemical/hormonal markers, cranial bone mineral density, biological sex, and mutation severity

**Fig 2:**
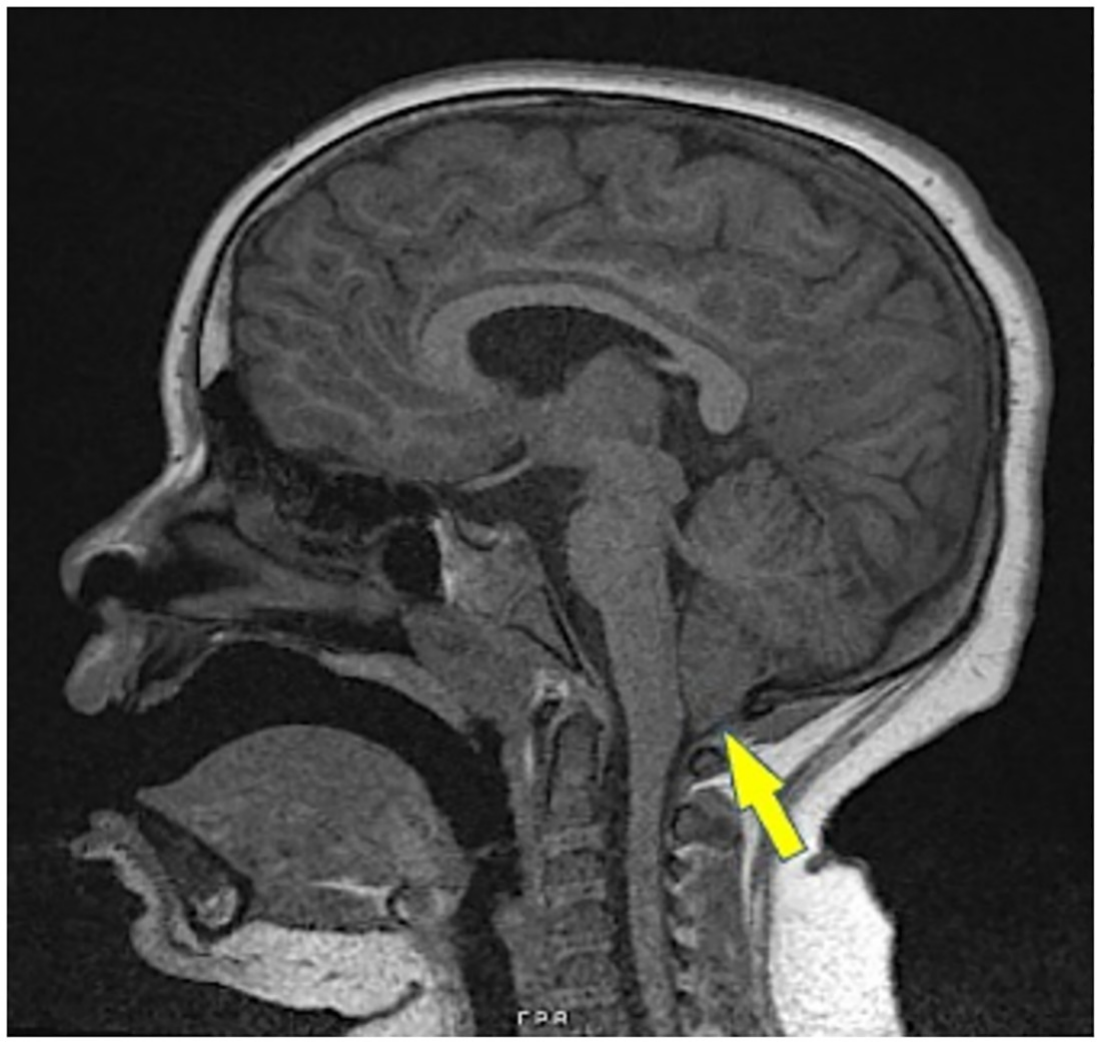
Brain MRI of PHP1A participant. Sagittal MRI image from participant #5. The cerebellar tonsils extend 7 mm below the foramen magnum reflecting Chiari type 1 malformation.

We first analyzed brain MRI and CT scans of a cohort of 54 PHP1A participants evaluated by our research team. All participants had a confirmed *GNAS* mutation with physical features (all confirmed by one consistent examiner, E.L.G-L.) and multi-hormone resistances consistent with PHP1A, with calcium, phosphorus, PTH, and thyroid function in good control at the time of GH testing (Table 1). Among the initial cohort of 54 participants, 8 were excluded from prevalence analysis due to a lack of an available MRI (Fig 1). When examining brain imaging within the remaining cohort, 5 of 46 participants (10.8%) were found to have CM1 on brain MRI as confirmed by a board-certified radiologist. Further analysis revealed that among these 5 participants, two were symptomatic prior to brain imaging. The first participant displayed findings of Horner syndrome on exam at approximately age 3 years; a head CT revealed hydrocephalus, and subsequent brain MRI imaging confirmed the presence of CM1. This participant later developed a decrease in vision in adolescence secondary to increased intracranial pressure and therefore had a second decompression surgery. The second participant complained of headaches near the time of GH testing and was subsequently found to have CM1 on MRI (Fig 2). The remaining 3 participants were asymptomatic prior to imaging. In addition to those patients with CM1, an additional 5 participants (10.8%) exhibited LLCT on imaging, which has been shown to be a pertinent finding in the development of CM1. Each of these 5 participants were asymptomatic prior to imaging. Within this group, all patients with the exception of one had a brain MRI performed after finding GH deficiency on stimulation testing, as per the accepted standard of care. Given that the prevalence of CM1 within the general population is estimated to be approximately 1 percent [30, 32, 33], our data suggest that PHP1A patients exhibit an approximately 10-fold increase in CM1 prevalence.

We next performed secondary analyses to assess whether we could identify independent factors that may contribute to the formation of CM1 and/or LLCT within PHP1A.

#### GH status

We first assessed whether there was a correlation between CM1 development and GH deficiency. Fig 1 delineates the inclusion criteria. Participants who were GH deficient were included only if the brain MRI was obtained around the time of GH stimulation testing or anytime thereafter. Participants who were GH sufficient were included regardless of the age at which the brain MRIs were obtained. These criteria led to inclusion of 43 participants, among whom 4 were GH sufficient and 39 were GH deficient. Despite the hypotheses suggested in previous case reports, we did not identify any significant correlation between GH deficiency and CM1 development in PHP1A (*p* = 0.18). In particular, 80.0% of PHP1A participants with either CM1 (4/5) or LLCT (4/5) were GH deficient, and 93.9% of unaffected PHP1A participants (31/33) were GH deficient.

#### Bone-related biochemical/hormonal markers

In addition to monitoring GH status, we assessed whether there was a correlation between CM1 and/or LLCT and bone formation markers including calcium, phosphorus, alkaline phosphatase, PTH, and 25-OH Vitamin D levels. We did not find any correlations with CM1 or LLCT when looking at these markers. Calcium and phosphorous levels were on average within normal limits. Alkaline phosphatase levels were normal for the participants’ ages. The majority of participants also had similar severity and duration of PTH elevations, with the exception of one participant. This participant did not have CM1 or LLCT, however she was noted to have significant swings in her PTH levels ranging from 14 to 636 pg/mL secondary to noncompliance. Her skull x-ray and head CT showed evidence of markedly increased hyperostosis.

#### Bone mineral density of the cranium

The participant mentioned above who had significant PTH swings and thickened cranium on skull x-ray and head CT had significantly elevated cranial BMD scores (mean 3.23 g/cm²) noted on a previously performed DXA scan. Therefore, we then looked at BMD scores of the cranium in a subset of participants with LLCT or in the group with neither CM1/LLCT who had DXA scans performed under a different research study [25]. In this small subset we did not find a correlation between LLCT and increased cranial BMD (*p* = 0.168) by Chi-square analysis.

#### Biological sex and *GNAS* mutation severity

No significant correlation between CM1 or LLCT with biological sex was found (*p* = 0.92). Additionally, there was no significant correlation to *GNAS* mutation severity (Table 1). Missense mutations are not considered severe in this analysis. For further evaluation, we also assessed whether there were specific mutations within *GNAS* that were associated with a greater chance of development of CM1. In particular, there is a common mutation in exon 7 (4 base pair *GNAS* deletion: c.565-568delGACT) that is a known susceptible area [88]. We did not find a correlation with this specific mutation and CM1 (*p* = 0.35).

### Chiari Type 1 malformation in PHP1A is not correlated with bone age advancement

We next assessed whether there was a correlation between CM1 or LLCT formation and bone age advancement. It is well known that patients with AHO can have marked advancement in their bone age x-ray, especially of the hand/wrist, secondary to premature chondrocyte differentiation. Available bone age radiographs of 37 participants were examined by one consistent examiner (E.L.G-L) as well as an independent radiologist. Eight had CM1 or LLCT, and 29 were unaffected. One patient with LLCT did not have a bone age but did have x-ray studies showing premature fusion of distal femurs by age 4. She was not included in the advanced bone age group resulting in a total of 36 participants for final analysis. From these studies, there was no significant difference (*p* = 0.34) in the extent of bone age advancement in participants with CM1/LLCT (2.33 ± 0.64 years) compared to participants without CM1/LLCT (1.62 ± 0.37 years). Chi square analysis also revealed no significant differences between the two groups in the percentage of participants with advanced bone ages of greater than or equal to 2 standard deviations above the mean as per the standard definition of an advanced bone age (*p* = 0.21).

In AHO patients the bone age advancement is typically more pronounced just before puberty and then increases at pubertal onset and as puberty progresses. It should be noted that the bone age x-rays of our participants were obtained at different ages depending on when participants were evaluated in our clinic and also the age of participants at the time of GH stimulation testing at which time a bone age was frequently obtained.

### Craniofacial abnormalities in AHO mouse model generated by *Gnas* exon 1 heterozygous inactivation

In order to understand further the etiologies surrounding the craniofacial malformations occurring in PHP1A, the craniofacial phenotype of our AHO mouse model (previously generated through targeted disruption of exon 1 of *Gnas* [22]) was examined. This model results in the global heterozygous inactivation of *Gnas*, and we have previously shown that this model phenotypically recapitulates many of the hormonal, metabolic, and skeletal abnormalities seen in patients with PHP1A and PPHP [22, 26, 68]. In particular, mice with maternally-inherited *Gnas* mutations (*Gnas E1+/-m*) phenotypically and hormonally recapitulate PHP1A; when compared to wildtype littermates, these mice have shortened lengths, are obese, develop subcutaneous ossifications [68], display hormonal resistance, and have decreased fertility [22]. Additionally, mice with paternally inherited *Gnas* mutations (*Gnas E1+/-p*) phenotypically resemble PPHP; *Gnas E1+/-p* mice have decreased length and develop subcutaneous ossifications but are not obese and do not display hormonal resistance or infertility [22, 68].

**Fig 3:**
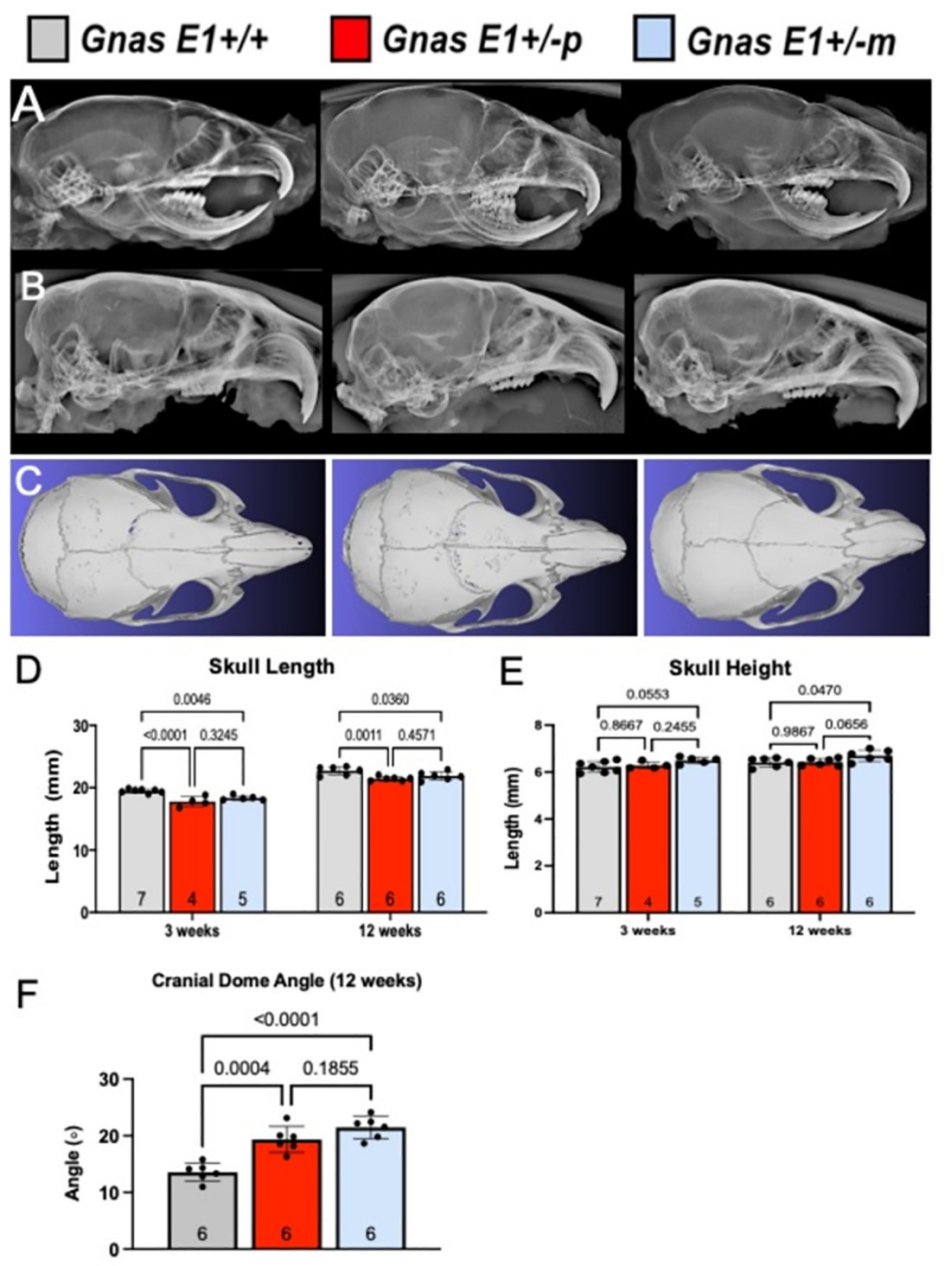
AHO mouse model generated by heterozygous inactivation of *Gnas* exon 1 (*Gnas E1+/-*) displays cranial hypoplasia. (A, B) Representative x-ray images of the skull of (A) 3 week and (B) 12-week-old *WT*, *Gnas E1+/-p* and *Gnas E1+/-m* mice. (C) Representative three dimensional reconstructions of the skull of 12-week-old *WT*, *Gnas E1+/-p* and *Gnas E1+/-m* mice. (D) Measurements of total skull length demonstrate that both *Gnas E1+/-p* and *Gnas E1+/-m* mice display a reduction in skull length at both 3 and 12 weeks of age when compared to *WT*. (E) Measurement of total skull height demonstrate no significant variations between *WT* and *Gnas E1+/-* mice at 3 weeks of age. *Gnas E1+/-m* mice at 12 weeks exhibited a mild but statistically significant increase in skull height when compared to *WT* mice. (F) Measurement of cranial dome angle demonstrates that both 12-week-old *Gnas E1+/-m* and *Gnas E1+/-p* mice exhibit significantly increased cranial dome angles when compared to *WT* mice. Sample size per genotype per experiment is listed on each graph. All statistical tests were performed by ANOVA with post-hoc Tukey tests for multiple comparisons, and *p*-values are displayed for each comparison.

We initially characterized the cranial architecture of *WT*, *Gnas E1+/-p* and *Gnas E1+/-m* mice at 3 weeks of age by x-ray and at 12 weeks by both x-ray and MCT (Fig 3A–3C). At each of these time points, skulls of both *Gnas E1+/-m* and *Gnas E1+/-p* mice appeared to have more rounded and dome-shaped structures when compared to *WT* mice (Fig 3A–3C). The skulls of both *Gnas E1+/-m* and *Gnas E1+/-p* mice were significantly shorter in length when compared to *WT* at both 3 and 12 weeks of age (Fig 3D). *Gnas E1+/-m* mice exhibited a mild but statistically significant increased cranial height when compared to *WT* mice at 12 weeks of age based on utilizing the measurements as described in Navein *et al*. [78]. No significant differences were observed between *WT* and *Gnas E1+/-m* mice at 3 weeks of age (Fig 3E) or between *WT* and *Gnas E1+/-p* mice at either 3 or 12 weeks of age (Fig 3E). In alignment with observations by x-ray imaging, further characterization of three-dimensional reconstructions of MCT scans of 12-week-old *Gnas E1+/-m* and *Gnas E1+/-p* mice exhibited an increased cranial dome angle when compared to *WT* mice (Fig 3F). Collectively, these data demonstrate that adult *Gnas E1+/-* mice exhibit craniofacial abnormalities, and the fact that these phenotypic changes occur in both *Gnas E1+/-m* and *Gnas E1+/-p* mice suggests that these abnormalities may be due to *Gnas* haploinsufficiency rather than being secondary to *Gnas* imprinting.

### Examination of the spheno-occipital synchondrosis in *Gnas E1+/-* mice

**Fig 4:**
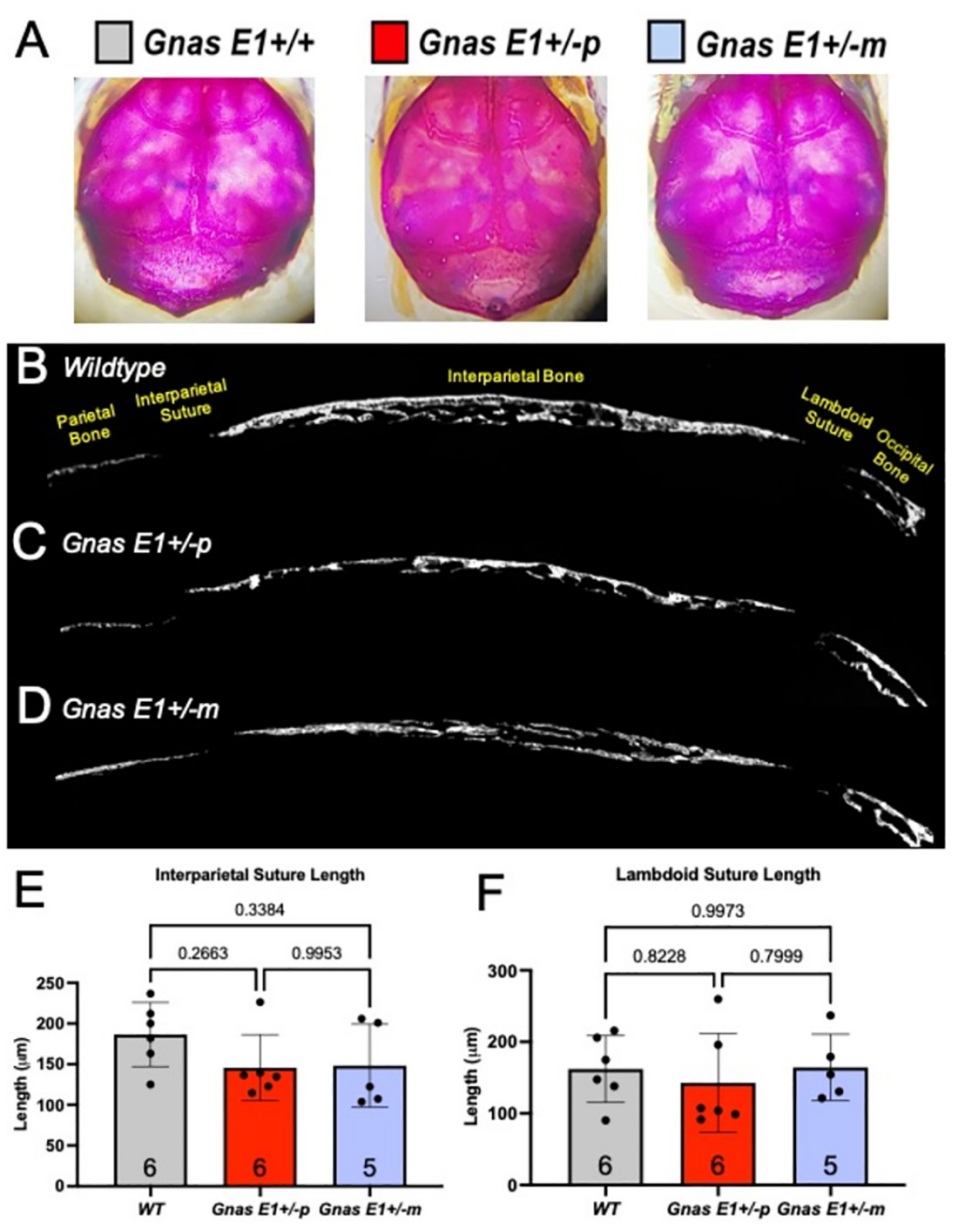
*Gnas* E1+/- mice do not display significant variations in the rate of interparietal or lambdoid suture closure. (A) Representative whole mount images of the cranium of 2-week-old WT, *Gnas* E1+/-p and *Gnas* E1+/-m mice. (B-D) Representative images of the cranium of 1-week- old (B) WT, (C) *Gnas* E1+/-p and (D) *Gnas* E1+/-m mice stained with calcein blue to visualize total mineral content and distance of interparietal and lambdoid sutures. (E-F) Quantification of the (E) Interparietal and (F) Lambdoid suture length demonstrated no significant differences between 1 week-old WT and *Gnas* E1+/- mice. Sample size per genotype per experiment is listed on each graph. All statistical tests were performed using a two-way ANOVA with post-hoc Tukey test for multiple comparisons, and *p*-values are displayed for each comparison.

**Fig 5:**
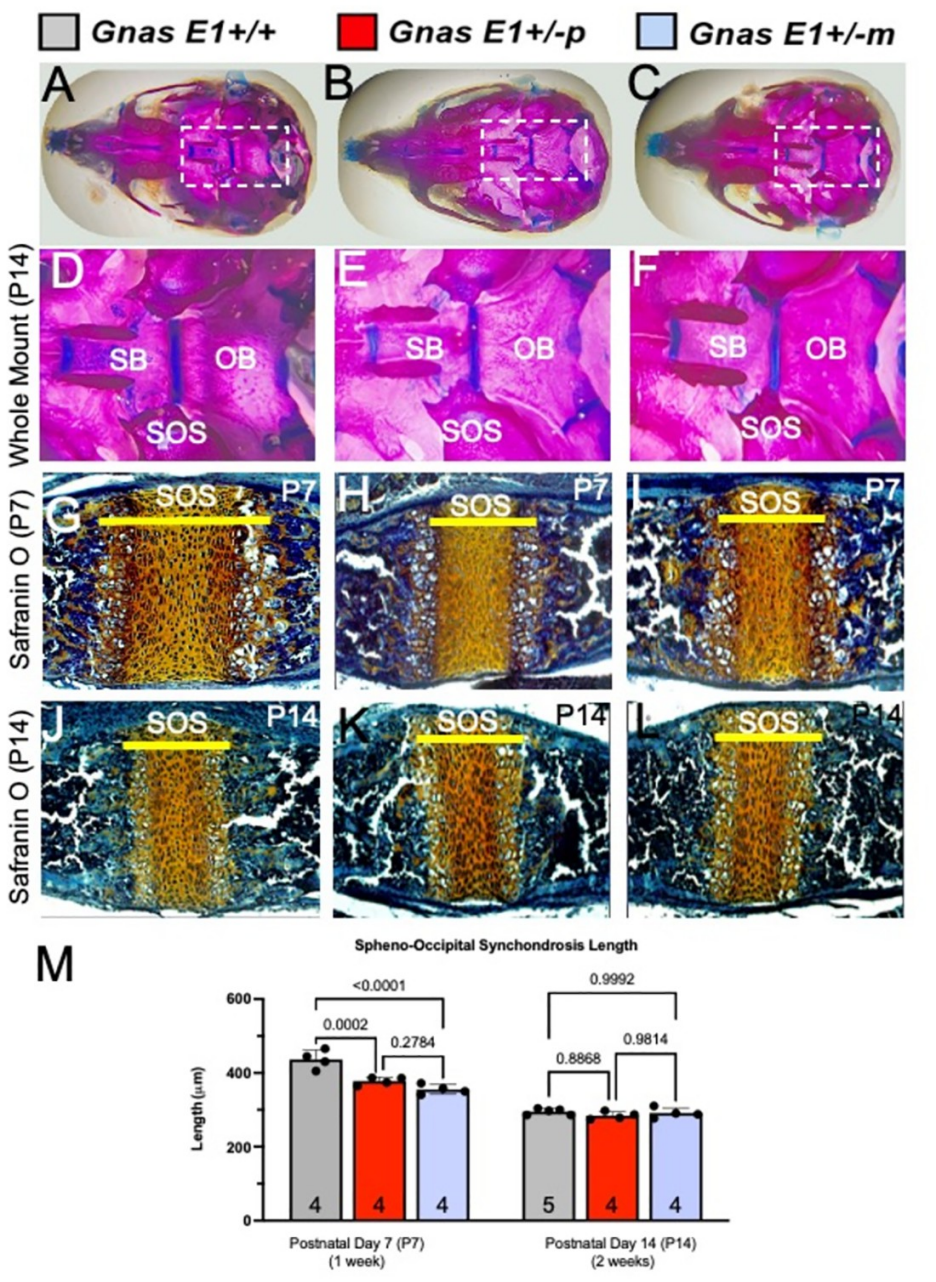
*Gnas* E1+/- mice display a significant reduction in spheno-occipital synchondrosis length. Representative low power (A-C) and higher power (D-F) whole mount images of the cranial base of 2-week-old (P14) (A, D) WT, (B, E) *Gnas* E1+/-p and (C, F) *Gnas* E1+/-m mice. Dashed rectangle in A-C is shown as larger image within D-F and highlights spheno-occipital synchondrosis. (G-I) Representative images of the cranial base of 1-week-old (P7) (G) WT, (H) *Gnas* E1+/-p and (I) *Gnas* E1+/-m mice stained with safranin O and fast green. (J-L) Representative images of the cranial base of 1-week-old (P7) (J) WT, (K) *Gnas* E1+/-p and (L) *Gnas* E1+/-m mice stained with safranin O and fast green. (M) Measurement of total SOS length (yellow line) among WT, *Gnas* E1+/-p and *Gnas* E1+/-m mice at both one and two weeks of age. Sample size per genotype per experiment is listed on each graph. All statistical tests completed using a two-way ANOVA with post-hoc Tukey test for multiple comparisons, and *p*-values are displayed for each comparison. For orientation, the sphenoid bone (SB) and occipital bone (OB) are identified on panels D-F for appropriate localization in relation to the SOS.

**Fig 6:**
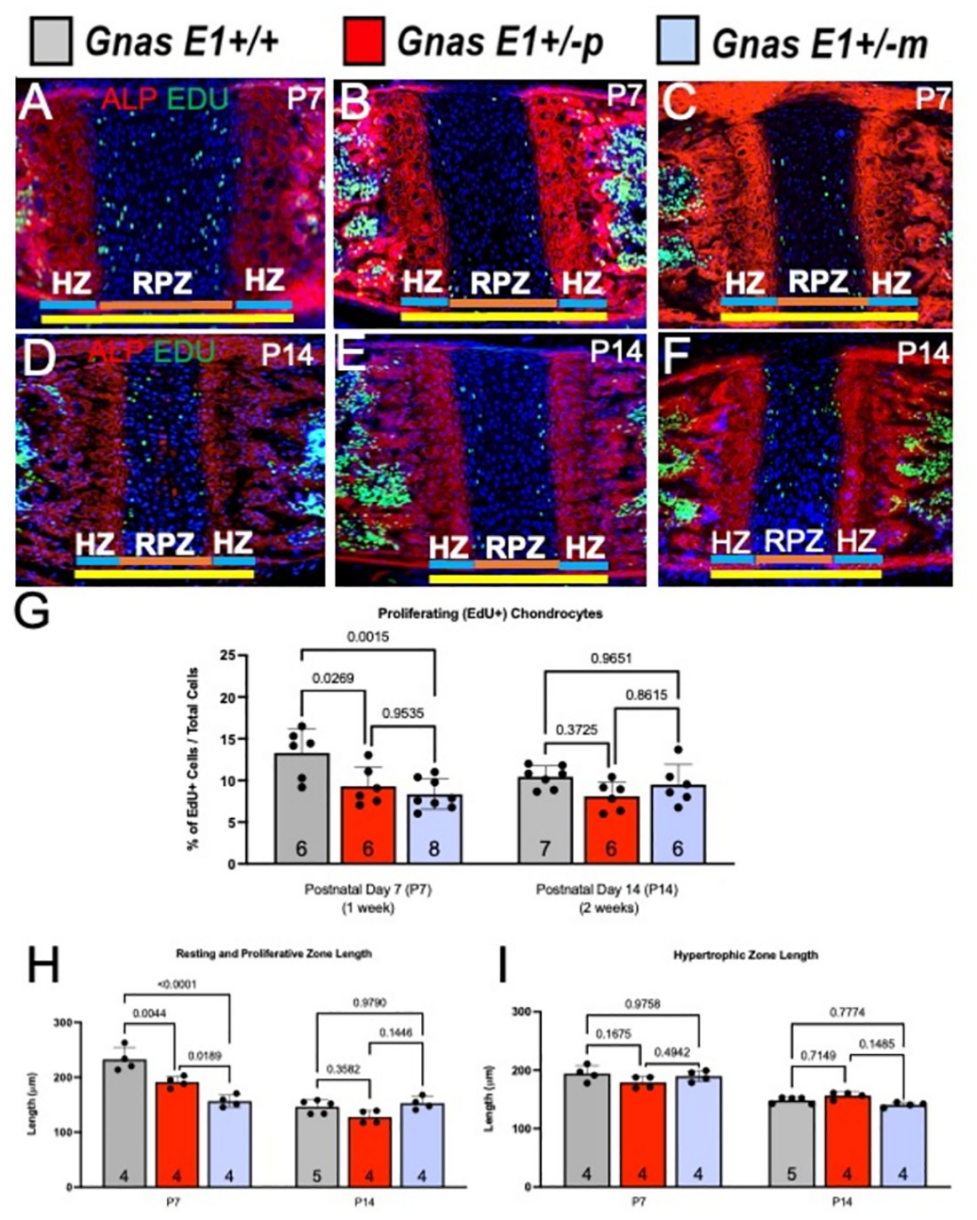
Accelerated reduction in spheno-occipital synchondrosis length within E1+/- mice is associated with a reduction in the number of proliferating chondrocytes. (A-F) Representative images of the SOS (yellow line) in (A-C) 1-week-old mice and (D-F) 2-week-old mice. In Fig 6 (A, D) are WT, (B, E) are *Gnas* E1+/-p, and (C, F) are *Gnas* E1+/-m mice stained for Alkaline phosphatase (red), EdU (green), and DAPI (blue). (G) Quantification of the percentage of EdU+ chondrocytes to the total number of chondrocytes within the resting and proliferative zone at 1 week of age demonstrates that both *Gnas* E1+/-p and *Gnas* E1+/-m mice display a significant reduction in proliferating chondrocytes compared to WT. No significant differences were observed at 2 weeks of age. (H) Quantification of the resting and proliferative zone lengths (defined as ALP negative zone of synchondrosis) (blue line) were significantly reduced in both *Gnas* E1+/-p and *Gnas* E1+/-m mice when compared to WT at 1 week of age. No significant differences were observed at 2 weeks of age. (I) Quantification of the hypertrophic zone length (defined as ALP+ zone of synchondrosis) (orange lines) demonstrated no significant differences between WT and *Gnas* E1+/- mice at 1 or 2 weeks of age. Sample size per genotype per experiment is listed on each graph. All statistical tests were completed using a two-way ANOVA with post-hoc Tukey test for multiple comparisons, and *p*-values are displayed for each comparison.

We next assessed whether *Gnas E1+/-* mice exhibit changes in cranial suture closure (Fig 4). Characterization of the cranial sutures at postnatal day 14 (P14) by whole mount staining did not reveal any evidence of craniosynostosis in *Gnas E1+/-* and *WT* mice (Fig 4A). Additionally, histomorphometric analysis of mineralized tissue within sagittal skull sections of postnatal day 7 (P7) *Gnas E1+/-* and *WT* mice using calcein blue staining demonstrated no significant differences in the overall length of the interparietal or lambdoid sutures (Fig 4B–4F). These data therefore suggest that the craniofacial abnormalities observed in both *Gnas E1+/-m* and *Gnas E1+/-p* mice are unlikely to be driven by premature cranial suture fusion. However, in addition to the cranial sutures, we examined the cranial base synchondroses of P7 and P14 *WT* and *Gnas E1+/-* mice (Fig 5). Whole mount staining of P14 mice identified that both *Gnas E1+/-m* and *Gnas E1+/-p* mice appeared to have a potential narrowing of the spheno-occipital synchondrosis (SOS) when compared to *WT* (Fig 5A–5F). For orientation, the sphenoid bone (SB) and occipital bone (OB) are also identified within Fig 5. Histologic analysis of the SOS of P7 (Fig 5G–5I) and P14 (Fig 5J–5L) mice revealed that at P7, *Gnas E1+/-m* and *Gnas E1+/-p* mice exhibit a significant reduction in SOS length when compared to *WT* (Fig 5M). This observation was of particular interest because the SOS is a structure recognized to be essential for craniofacial development, and accelerated closure of this structure in humans and mice have been correlated with the development of midface hypoplasia [52]. Therefore, we performed histomorphometric analysis of the SOS in P7 and P14 *WT* and *Gnas E1+/-* mice by utilizing a combination of both EdU pulse-chasing to label proliferative chondrocytes and Alkaline Phosphatase (ALP) histochemistry to label hypertrophic chondrocytes (Fig 6A–6F). We did not observe any significant differences in SOS length, numbers of EdU+ chondrocytes, resting and proliferative zone length, or hypertrophic zone length between P14 *Gnas E1+/-* and *WT* mice (Figs 5M, 6G–6I). Examination of P7 mice, however, revealed that the shortened SOS length in both *Gnas E1+/-m* and *Gnas E1+/-p* mice was directly associated with a reduction in the total percentage of EdU+ chondrocytes within the SOS (Fig 6G) as well as a significant reduction in the SOS resting and proliferative zones (Fig 6H) when compared to *WT* mice. We did not observe any significant differences in the length of the SOS hypertrophic zone between P7 *Gnas E1+/-* and *WT* mice (Fig 6I). Therefore, these data suggest that the observed craniofacial abnormalities observed in *Gnas E1+/-* mice may be associated with abnormal skeletal patterning secondary to premature closure of the SOS due to accelerated proliferative chondrocyte differentiation.

### Cranial hyperostosis and enhanced cranial bone formation in *Gnas E1+/-m* mice

**Fig 7:**
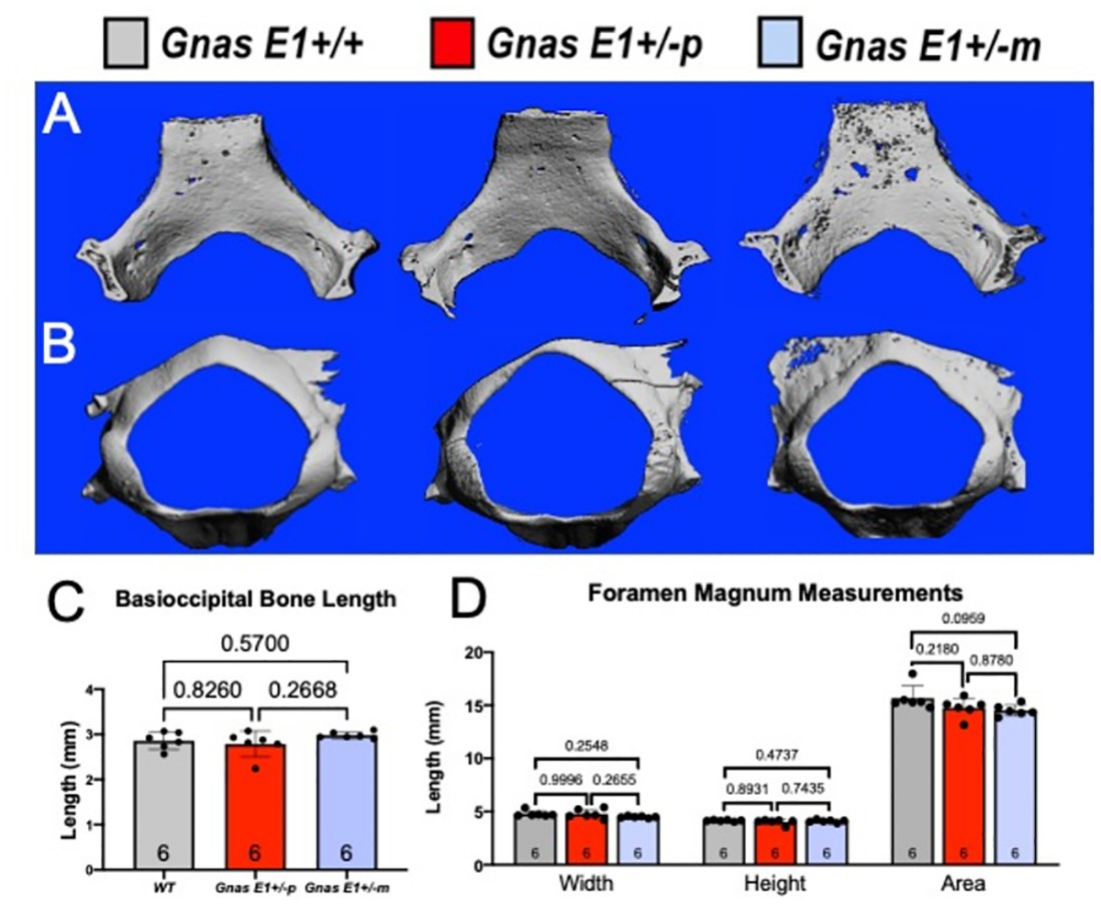
*Gnas* E1+/-m mice exhibit a reduction in foramen magnum width and area. (A, B) Representative three-dimensional reconstructions of the (A) basioccipital bone and (B) foramen magnum of 12-week old WT, *Gnas* E1+/-p and *Gnas* E1+/-m mice. (C) Measurements of basioccipital bone length in 12-week WT and *Gnas* E1+/- mice demonstrate no significant differences. (D) Measurement of foramen magnum width, height, and area demonstrate that *Gnas* E1+/-m and *Gnas* E1+/-p mice display no significant differences in foramen magnum width, height or total area when compared to WT mice. Sample size per genotype per experiment is listed on each graph. All statistical tests were completed using ANOVA with post-hoc Tukey test for multiple comparisons, and *p*-values are displayed for each comparison.

**Fig 8:**
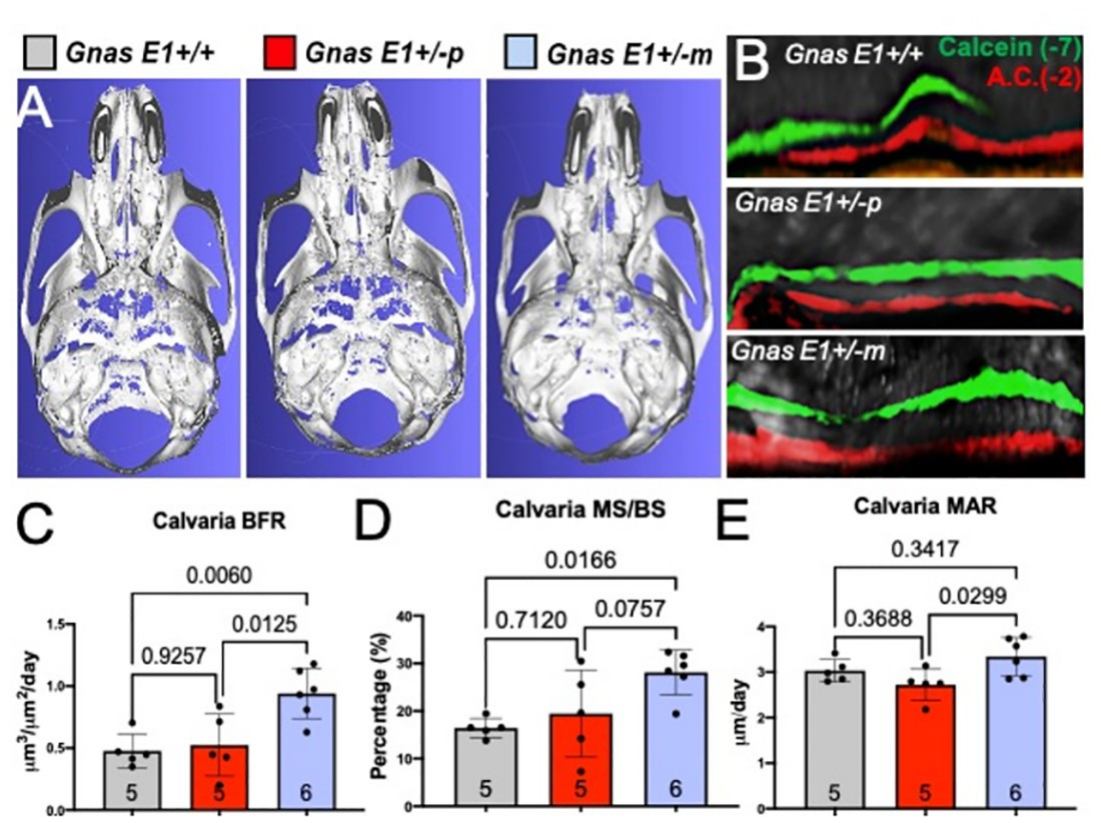
*Gnas* E1+/-m mice display cranial hyperostosis and enhanced calvarial bone formation *in vivo*. (A) Representative 3D reconstruction of the cranial vault of 12-week WT, *Gnas* E1+/-p, and *Gnas* E1+/-m mice demonstrating hyperostosis within *Gnas* E1+/-m mice. (B) Representative calcein and alizarin complexone double labeling on the calvaria of 12-week WT, *Gnas* E1+/-p, and *Gnas* E1+/-m mice. (C-E) Quantification of (C) Bone formation rate (BFR); (D) mineralizing surface to bone surface (MS/BS); and (E) Mineral apposition rate (MAR) within the calvaria demonstrates *Gnas* E1+/-m mice display enhanced bone formation when compared to both WT and *Gnas* E1+/-p mice. No significant differences were observed between WT and *Gnas* E1+/-p mice. Sample size per genotype per experiment is listed on each graph. All statistical tests were completed using ANOVA with post-hoc Tukey test for multiple comparisons, and *p*-values are displayed for each comparison.

Given the cranial base abnormalities that we observed in *Gnas E1+/-* mice, we next wanted to assess whether these findings could shed light on the elevated prevalence of CM1 and LLCT in patients with PHP1A. We first addressed whether 12-week-old *Gnas E1+/-* mice have structural changes within the occipital bone of the cranial base (Fig 7). We initially examined the occipital bone because its multiple subcomponents (including the basioccipital, squamous, and lateral portions) serve an essential role in the formation of the cranial base, posterior fossa, and foramen magnum, and aberrant development of these components has been identified as a potential contributing cause of CM1 [89, 90]. We therefore compared the basioccipital bone length and the foramen magnum structures of 12-week *WT* and *Gnas E1+/-* mice by MCT (Fig 7A and 7B), and through these analyses we did not observe any significant differences in basioccipital bone length (Fig 7C) or in the width, height, or area of the foramen magnum (Fig 7D). Due to the lack of structural changes of the occipital bone, we next evaluated the cranial bone architecture of 12-week-old *WT* and *Gnas E1+/-* skull MCT images that were three dimensionally (3D) reconstructed (Fig 8A). From these evaluations, we consistently observed that *Gnas E1+/-m* mice exhibit evidence of hyperostosis within the cranium and cranial base when compared to both *WT* and *Gnas E1+/-p* mice (Fig 8A). These data were of particular interest given that we have previously shown *Gnas E1+/-m* mice to display significantly elevated serum P1NP and histologically to exhibit enhanced bone formation when compared to *WT* and *Gnas E1+/-p* mice. C-telopeptide levels were increased in female *Gnas E1+/-p* mice, but there were no significant changes in *Gnas E1+/- mice*. [26]. Furthermore, previous reports have described the presence of a thickened cranium on head CT imaging of PHP1A patients [11, 58, 91] as well as increased bone mineral density in PHP1A [25]. We therefore performed dynamic histomorphometry to assess bone formation rate (BFR) within both the calvaria (Fig 8B–8E) and basioccipital bones (S1 Fig) of 12-week-old mice following intraperitoneal injections of calcein and alizarin complexone bone mineral labels. Calvarial histomorphometry revealed *Gnas E1+/-m* mice display a significant increase in bone formation rate when compared to both *WT* and *Gnas E1+/-p* mice (Fig 8B and 8C). Furthermore, *Gnas E1+/-m* mice display a significantly elevated ratio of mineralizing surface to bone surface (MS/BS) when compared to *WT* mice; there were no statistically significant differences in MS/BS between *Gnas E1+/-m* and *Gnas E1+/-p* mice (Fig 8D). Additionally, *Gnas E1+/-m* mice have a significant increase in mineral apposition rate (MAR) when compared to *Gnas E1+/-p* mice but no significant differences in MAR when compared to *WT* (Fig 8E). No significant differences in BFR, MS/BS, or MAR were observed between *WT* and *Gnas E1+/-p* mice (Fig 8B–8E). Dynamic histomorphometry of the basioccipital bone revealed that *Gnas E1+/-m* and *WT* mice have no significant differences in BFR, MS/BS, or MAR (S1 Fig). We did, however, observe that *Gnas E1+/-p* mice display a significant reduction in basioccipital BFR and MS/BS when compared to both *WT* and *Gnas E1+/-m* mice (S1A–S1D Fig). In summary, these data provide direct evidence of increased bone formation within the cranium of *Gnas E1+/-m* mice when compared to *WT* and *Gnas E1+/-p* mice and suggest that this enhanced activity in conjunction with abnormal skeletal patterning due to premature SOS closure could potentially contribute to the presence of CM1 or low lying tonsils due to a reduction in cranial volume.

## Discussion

Our study is the first to identify an increased prevalence of CM1 and LLCT in a large cohort of mutation-confirmed PHP1A patients. Through this study we discovered that there is at least a 10-fold higher prevalence of CM1 in PHP1A compared to the general population. If LLCT is included, this prevalence increases to over 20-fold when compared to the general population. Identification of an increased prevalence of CM1 in PHP1A has important implications, including the need for earlier screening, close monitoring, and possible intervention even with mild symptoms in order to prevent serious outcomes such as hydrocephalus or syringomyelia formation. In addition, our data suggest that if a brain MRI were to be done for any clinical reason for a patient with PHP1A, it is important that it be specifically evaluated for the presence of CMI or LLCT whether or not there are clinical signs or symptoms since these can be subtle.

When evaluating for various independent risk factors, we did not observe any correlation between the frequency of CM1 or LLCT development and biological sex, *GNAS* mutation severity, bone age advancement, or GH status. Our findings regarding the lack of correlation to GH status are intriguing in light of a collection of isolated case reports that hypothesized GH deficiency as a possible cause of CM1 in PHP1A [57, 60], as well as numerous reports suggesting that GH deficiency plays a role in CM1 formation in the general population [47, 48]. We acknowledge that there may be sampling bias in our investigation given that 75% of our participants had brain MRIs performed secondary to GH deficiency on stimulation testing (as part of standard of care). This limited our ability to assess for CM1 or LLCT in those who are GH sufficient, as they do not have the clinical indication for obtaining brain MRI evaluations as do those who are GH deficient. Hence, the identification of CM1 or LLCT in additional GH-sufficient PHP1A patients would further validate our conclusion that GH deficiency does not contribute to the formation of CM1 or LLCT.

We further examined *Gnas E1+/-* mice in order to search for a potential mechanism by which *Gnas* inactivation affects cranial development. Previous studies have examined the role of lineage-specific homozygous deletion of *Gnas* in early mesenchymal progenitors (*Gnas*^*fl/fl*^*;Prrx1-CRE*) [92] and neural crest cells (*Gnas*^*fl/fl*^*;Wnt1-CRE*) [93] and concluded that the craniofacial abnormalities associated with *Gnas* homozygous deletion are secondary to accelerated osteogenesis within the cranium resulting in craniosynostosis, a condition occurring occasionally in PHP1A [2, 10, 60, 93, 94]. Through histologic analysis of one-week-old mice, we did not observe significant differences in the closure of interparietal or lambdoid sutures in *Gnas E1+/-* mice when compared to *WT*. However, we observed that these *Gnas E1+/-* mice exhibited a significant closure of the SOS when compared to *WT*. This observation was of particular interest because the SOS is a cartilaginous structure that serves an essential role in the development of the cranial base, and accelerated closure of the SOS in humans and mice has been correlated with aberrant cranial patterning and the development of midface hypoplasia [52]. Furthermore, a collection of reports has implicated similarities between cranial synchondroses and the epiphyseal plates of long bones due to the cell types harboring these regions, as well as the presence of highly conserved signaling pathways influencing cellular differentiation and bone development [53, 95, 96].

Although midface hypoplasia is a common finding in our hundreds of mutation-confirmed PHP1A patients, we did not find a correlation of midface hypoplasia with CM1 or LLCT in our current cohort investigated in this study. Midface hypoplasia is not always apparent early in life, therefore making the true correlation difficult to assess. Clinically, however, it is important to review our PHP1A patient population at large for those who have midface hypoplasia, and further investigations are underway for more precise characterization in humans to look for a possible correlation between midface hypoplasia and CM1/LLCT.

Our investigations of the SOS in one-week and two-week-old *WT* and *Gnas E1+/-* mice revealed accelerated proliferative chondrocyte differentiation as the cause of premature closure of the SOS. This correlates with clinical findings in AHO patients of rapidly advancing bone ages and premature chondrocyte differentiation at the epiphyses, especially of the hand/wrist [1, 3, 4, 9, 17, 64–66]. Given these findings clinically and in our mouse model of the premature closure of the SOS, our data suggest that this premature fusion may play a role in the cranial abnormalities observed in PHP1A. Our study, however, did not reveal a correlation between bone age advancement and CM1/LLCT formation. There are a number of potential possibilities to explore in this regard for the lack of correlation. One potential possibility could be because the bone age x-rays were obtained at different ages depending on the time of evaluation in our clinic and age at GH stimulation testing (a common point at which to assess bone age). In AHO patients, the bone age advancement is typically more pronounced just prior to puberty and at pubertal onset, with an acceleration of bone age as puberty progresses [4, 9, 17]. Since this bone age advancement may not be apparent until the patient is closer to pubertal onset, we speculate that if we had bone ages available for all patients near or at the time of puberty, we could potentially find a correlation between advanced bone age and CM1/LLCT. Unfortunately, not all patients had reached pubertal age in our study. Future investigations of bone ages in our cohort over time as the younger participants get closer to puberty are warranted in order to assess whether bone age could indeed be a “biomarker” to assess for a greater risk for CM1/LLCT formation. Finally, a larger cohort of patients for analysis could provide more insight into our bone age analyses. Although the number of patients are relatively large for a rare condition, the sample size is still small overall for correlation analyses.

Another potential cause of CM1 formation includes the small cranial vault typical of PHP1A thought to be secondary to cranial hyperostosis [54]. Examination of the cranium in our mouse model revealed that both *Gnas E1+/-m* and *Gnas E1+/-p* mice appear to have a shortened cranium as well as increased cranial height and cranial dome angle when compared to *WT* mice. We also found that *Gnas E1+/-m* mice have evidence of hyperostosis within the cranium and cranial base when compared to both *WT* and *Gnas E1+/-p* mice. This finding is in alignment with clinical reports of cranial thickening seen on radiography in PHP1A patients [11], including patients followed in our clinic. Because either X-ray or CT imaging is necessary for adequate assessment of cranial thickening [97], we were unable to do a correlation study in our participants with brain MRI evaluations. However, clinically, we do have patients who had either x-rays or head CT imaging performed who have been noted to have thickened craniums, but we did not have a cohort large enough in this study to assess this feature adequately, as radiographic and CT imaging are only performed when clinically necessary.

In order to assess this more fully, we reviewed the various bone markers over time in each patient examined, such as serum calcium, phosphorous, alkaline phosphatase, PTH, and 25-OH Vitamin D levels. We did not find any correlations with CM1 or LLCT when looking at these markers. The majority of patients also had a similar severity and duration of PTH elevations, thus making it difficult to investigate correlations. Additionally, we examined available prior DXA reports for cranial BMD measurements. Unfortunately, we did not have DXA results for any of the participants with CM1; however we did have several cranial DXA scans for participants with LLCT and in the group with neither (performed in a prior published study [25]). We did not find a correlation between LLCT and increased cranial BMD. Interestingly, there was one female participant without CM1 or LLCT who did have both a head CT as well as cranial DXA imaging that exhibited cranial hyperostosis. Her head CT revealed an extremely thickened cranium, which correlated with her significantly elevated cranial BMD on DXA. We hypothesize that this may be secondary to her longstanding wide fluctuations in PTH levels due to non-compliance with her calcitriol, thereby causing hyperostosis, similar to the mechanism by which parathyroid hormone analogs induce bone formation. In our mouse model of PHP1A we found that this increased BMD is most likely secondary to altered Gαs signaling to a large degree [26], but this does raise the possibility that the hyperostosis could additionally be related to marked PTH fluctuations. Further experiments in mice and studies in patients would be ideal to explore this potential correlation and its potential association with CM1/LLCT formation. Our prior findings demonstrated that PHP1A patients have normal to increased bone mineral density in the whole body and lumbar spine [25]. These findings also correlated with our findings in *Gnas E1+/-m* mice, which display enhanced bone parameters due to both increased osteoblast activity and normal bone resorption, leading to an overall increase in bone formation [4, 26, 27]. This suggests that there is a generalized increase in bone formation in the cranium as well. Together these findings demonstrate that *Gnas E1+/-* mice have aberrant skull formation that may cause overall decreased volume in the cranium, potentially increasing the risk for CM1 and LLCT formation.

Our investigations here have focused primarily on the PHP1A subtype of AHO, and we do not know if PPHP patients have an increased prevalence of CM1. Clinically these patients do not have hormonal resistance and therefore are not GH deficient due to GHRH resistance. They have no usual indications for brain imaging and could be asymptomatic, thus making it difficult to investigate the prevalence of CM1 in this population. They do, however, have the same bone phenotype in terms of skeletal abnormalities and advanced bone ages [1, 4, 9, 17, 67, 77]. In our current mouse studies, we observed a similar shortening of the SOS in *Gnas E1+/-p* mice; however, our prior studies showed no increase in bone formation rates as had been seen for *Gnas E1+/-m* mice [4, 26, 27]. Further work in this area is needed in order to investigate the relationship between CM1/LLCT and PPHP.

Our findings have important clinical implications for patients with PHP1A. With an increased prevalence of CM1 and LLCT, it is important to monitor patients with PHP1A closely for potential signs and symptoms, particularly given that surgical interventions can improve outcomes and decrease morbidity and mortality in many cases [35, 37, 98]. Additionally, given that nearly 70% of PHP1A patients develop GH deficiency [4, 9, 17, 18] and are treated with GH, it is important to distinguish headache caused by pseudotumor cerebri, a potential rare side effect of GH therapy, from headache caused by CM1. In rare instances in which pseudotumor cerebri is considered, we also urge clinicians to consider the possibility of CM1 or LLCT. In addition, in any instance in which a PHP1A patient has brain imaging, we recommend careful examination for the possible presence of CM1 or LLCT. With early recognition and monitoring, adverse outcomes may be avoided.

## Supporting information

S1 Fig Dynamic mineral labeling histomorphometry of Gnas E1+/- and WT basioccipital bone. (A) Representative calcein and alizarin complexone double labeling within the basioccipital bone of WT, Gnas E1+/-p and Gnas E1+/-m mice. (B-D) Quantification of (B) BFR, (C) MS/BS and (D) MAR within the basioccipital bone demonstrate that Gnas E1+/-m mice display normal bone formation when compared to WT, whereas Gnas E1+/-p mice display a reduction in BFR when compared to both WT and Gnas E1+/-m mice. Sample size per genotype per experiment is listed on each graph. All statistical tests completed using ANOVA with post-hoc Tukey test for multiple comparisons, and p-values are displayed for each comparison.
(TIF)
