# Supplementary figures and images for: Prevalence of Chiari malformation type 1 is increased in pseudohypoparathyroidism type 1A and associated with aberrant bone development
Source: PLoS One. 2023 Jan 20;18(1):e0280463. doi: 10.1371/journal.pone.0280463 (PMC9858345; doi:10.1371/journal.pone.0280463)

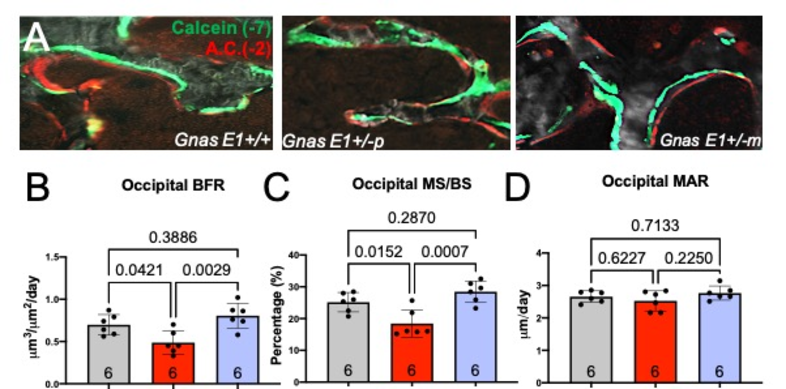

Supplement: S1 Fig — (A) Representative calcein and alizarin complexone double labeling within the basioccipital bone of WT, Gnas E1+/-p and Gnas E1+/-m mice. (B-D) Quantification of (B) BFR, (C) MS/BS and (D) MAR within the basioccipital bone demonstrate that Gnas E1+/-m mice display normal bone formation when compared to WT, whereas Gnas E1+/-p mice display a reduction in BFR when compared to both WT and Gnas E1+/-m mice. Sample size per genotype per experiment is listed on each graph. All statistical tests completed using ANOVA with post-hoc Tukey test for multiple comparisons, and p-values are displayed for each comparison. (TIF) [file pone.0280463.s001.tif]
